# I21: an advanced high-resolution resonant inelastic X-ray scattering beamline at Diamond Light Source

**DOI:** 10.1107/S1600577522000601

**Published:** 2022-02-22

**Authors:** Ke-Jin Zhou, Andrew Walters, Mirian Garcia-Fernandez, Thomas Rice, Matthew Hand, Abhishek Nag, Jiemin Li, Stefano Agrestini, Peter Garland, Hongchang Wang, Simon Alcock, Ioana Nistea, Brian Nutter, Nicholas Rubies, Giles Knap, Martin Gaughran, Fajin Yuan, Peter Chang, John Emmins, George Howell

**Affiliations:** a Diamond Light Source, Harwell Campus, Didcot OX11 0DE, United Kingdom

**Keywords:** synchrotron radiation, soft X-ray beamline, resonant inelastic X-ray scattering, VLS-PGM, spectrometer

## Abstract

The I21 beamline at Diamond Light Source is dedicated to advanced resonant inelastic X-ray scattering (RIXS) for probing charge, orbital, spin and lattice excitations in materials across condensed matter physics, applied sciences and chemistry. Both the beamline and the RIXS spectrometer employ divergent variable-line-spacing gratings covering a broad energy range of 280–3000 eV. A combined energy resolution of ∼35 meV (16 meV) is readily achieved at 930 eV (530 eV) owing to the optimized optics and the mechanics. Considerable efforts have been paid to the design of the entire beamline, particularly the implementation of the collection mirrors, to maximize the X-ray photon throughput. The continuous rotation of the spectrometer over 150° under ultra high vacuum and a cryogenic manipulator with six degrees of freedom allow accurate mappings of low-energy excitations from solid state materials in momentum space. Most importantly, the facility features a unique combination of the high energy resolution and the high photon throughput vital for advanced RIXS applications. Together with its stability and user friendliness, I21 has become one of the most sought after RIXS beamlines in the world.

## Abstract 2

The design of the resonant inelastic X-ray scattering beamline at Diamond Light Source, I21, is presented. X-ray commissioning results are shown and compared with the optical simulations.

## 1. Introduction

Resonant inelastic X-ray scattering (RIXS) is a powerful spectroscopic technique capable of probing charge-neutral excitations, such as charge-transfer excitations (*e.g.* between the ligand anions and the metal cations), and orbital excitations (*e.g.* *dd* or *ff*), in a broad range of materials. With an improved energy resolution corresponding to the mid-infrared energy regime (*i.e.* 100–500 meV), RIXS, in particular the direct RIXS at the *L*-edges of transition metal elements, has shown to be capable of characterizing collective magnetic excitations in quantum magnetic materials (Braicovich *et al.*, 2009 ▸; Ament *et al.*, 2011 ▸). This newly revealed capability has excited considerable interest in the condensed matter physics community due to its complementarity with inelastic neutron scattering and because it is particularly advantageous for probing magnetic excitations in small samples or complex systems with multiple magnetic elements. RIXS has thus become a sought after technique, and, as such, the new generation of soft X-ray RIXS instruments (*e.g*. ID32 at ESRF, I21 at DLS, SIX at NSLS II, AGM-AGS at TPS, VERITAS at MAX IV) have been proposed and constructed, aiming to achieve a resolution of ∼25 meV comparable with *k*_B_ *T* at ambient temperature (Brookes *et al.*, 2018 ▸; Dvorak *et al.*, 2016 ▸; Singh *et al.*, 2021 ▸).

Among the newly delivered advanced RIXS instruments, the I21-RIXS beamline has achieved a robust combined energy resolution of ∼35 meV at the Cu *L*-edge, and ∼16 meV at the O *K*-edge. The breakthrough of the energy resolution has led to a wave of novel discoveries. For instance, charge density wave (CDW) excitations, electron–phonon anomalies, and acoustic plasmons were revealed in various cuprate superconductors (Li *et al.*, 2020 ▸; Lee *et al.*, 2021 ▸; Lin *et al.*, 2020 ▸; Nag *et al.*, 2020 ▸). For the newly discovered infinite-layer nickelate superconductors, RIXS revealed remarkable dispersive magnons akin to that of cuprates despite crucial differences in the electronic structures of the two (Lu *et al.*, 2021 ▸). With the improved energy resolution, new insights were obtained on the orbiton physics in a canonical orbital system KCuF_3_ (Li *et al.*, 2021 ▸). In material sciences, a coherent many-body exciton was discovered in a van der Waals antiferromagnet (Kang *et al.*, 2020 ▸). I21 has also provided crucial spectroscopic evidence in identifying oxidized O_2_ trapped in the highly charged unstable Li-rich battery cathode materials (House *et al.*, 2020*a* ▸,*b* ▸).

Besides the research at the *L*-edges of 3*d* transition metal oxides (TMOs), there has been mounting interest in applying RIXS to 4*d* and 5*d* TMOs (Gretarsson *et al.*, 2020 ▸; Moretti Sala *et al.*, 2018 ▸). Therefore, the I21 beamline was proposed to deliver a dedicated high-resolution RIXS facility with a broad energy range from 280 eV to 3000 eV covering the C *K*-edge, O *K*-edge, all 3*d* *L*-edges, most 4*d* *L*-edges and some 5*d* *M*-edges.

To obtain high energy resolution in the soft X-ray range, high-quality grating optics are essential as they contribute directly to the energy broadening. Also, a long instrument is strongly desirable given all energy-resolving optics work at grazing angles. We expand the discussions of how to achieve a high-resolution soft X-ray RIXS beamline and spectrometer in Section 2. On the other hand, improving the energy resolution means that the X-ray photon throughput will be naturally reduced. Therefore a long undulator source, a simple beamline with minimal number of optics are preferable. For the spectrometer, collecting optics are imperative to enhance the acceptance angle of the high-resolution RIXS. Details of how we achieve a high photon throughput are laid out in Section 3. To target RIXS applications in three-dimensional materials, a complex cryogenic manipulator with six degrees of freedom is implemented for the momentum space mapping (see Section 4). The general mechanical design of the RIXS spectrometer is also presented in Section 4. To make the very high energy resolution practically usable, the whole instrument needs to be stable and user friendly. We discuss the relevant design and achievements including the long-term stability of the beamline in Section 5.

## 2. The energy resolution of the I21 RIXS beamline

### 2.1. Optical scheme of the beamline and the spectrometer

The I21 beamline employs the divergent variable line spacing (dVLS) plane-grating optical design to achieve the best possible energy resolution at the soft X-ray energy range. This was corroborated by a thorough investigation of the dVLS optics scheme together with the collimated constant line spacing plane-grating monochromator (cPGM) and the convergent variable line spacing (cVLS) plane-grating monochromator optical designs (Strocov *et al.*, 2010 ▸; Pereiro *et al.*, 2009 ▸). As detailed in Section 2.2, the pre-mirror and gratings in the PGM are the main optics contributing to the energy resolution in the dVLS concept. However, in the cPGM or cVLS scheme, extra components such as the collimating or (re)focusing mirrors affect the energy resolution. In addition, these optics also reduce the total efficiency of the beamline and make the alignment of the beamline more complex. These are detrimental factors to the photon-hungry RIXS technique, especially when a very high energy resolution and precision alignment are desired.

For the RIXS spectrometer, the most commonly implemented optics schemes include the Rowland circle design (Nordgren *et al.*, 1989 ▸), the VLS-Hettrick Underwood plane-grating design (Hettrick *et al.*, 1988 ▸), the AGM-AGS active grating design (Lai *et al.*, 2014 ▸) and the spherical VLS (SVLS) grating design (Ghiringhelli *et al.*, 2006 ▸). We will not provide a systematic comparison of all optical schemes as this has been done previously (Dvorak *et al.*, 2016 ▸). Nevertheless, the same criteria hold for the selection of the optics scheme of the I21 RIXS spectrometer. We are strongly in favour of the SVLS concept based on the viewpoints that: (1) it is probably the simplest optical design as only one optics element, *i.e.* the SVLS grating, is in the dispersive plane. So in theory, for a given grating, the scheme could attain the best possible energy resolution than, for instance, the Hettrick Underwood design, in which more than one optics element is involved in the energy-resolved plane; (2) the optimization of the SVLS spectrometer for any energy is much less stringent than other schemes as one can find a focal condition by varying any of the entrance arm, the exit arm, or the grating incidence angle; (3) the focal plane can be optimized to a near grazing incident angle. For standard CCD detectors having an effective spatial resolution of 24 µm (Ghiringhelli *et al.*, 2006 ▸) (see the expanded discussion in Section 2.2), the tilted focal plane can significantly improve its effective spatial resolution.

**Figure 1:**
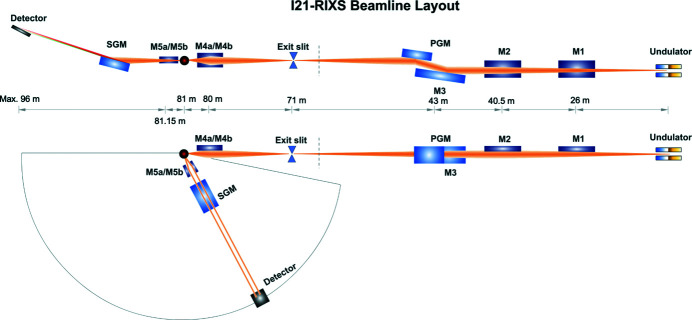
I21 beamline layout. The upper part is the side view while the lower part is the top view of the beamline layout. The distance of each component to the source of the undulator is labelled in the middle. The vertical bar upstream of the exit slit represents the partition between the internal and external beamline.

We show the entire I21 beamline optics layout in Fig. 1 ▸. X-rays generated from the APPLE-II undulator are fully accepted by the first plane optic M1. The M1 mirror acts to cut out high-energy radiation hence a lot of the radiation power of the X-ray beam. The tangentially cylindrical and sagittally plane M2 mirror focuses the horizontal X-ray beam to the exit slit. Deflected X-rays are dispersed by the dVLS plane-grating monochromator (PGM). The dVLS PGM comprises an internally water-cooled plane pre-mirror (M3) and four VLS plane gratings (VPG) which have water-cooled copper blocks clamped to the sides of the gratings. The X-rays are monochromated and vertically focused by the VLS grating to the exit slit. Thus monochromatic X-rays form a stigmatic source at the exit slit. The divergent X-rays are finally refocused by the M4 ellipsoidal mirror to the sample position. X-rays scattered from the sample are vertically intercepted by the SVLS grating delivering a monochromatic and converging beam focused onto the CCD detector. Horizontally two M5 plane-parabolic mirrors feature a very large horizontal acceptance angle hence relatively high photon throughput for the spectrometer. Detailed optical parameters of all beamline mirrors are summarized in Tables 1 ▸ and 2 ▸. The undulator parameters are shown in Table 3 ▸.

### 2.2. The energy resolution of the primary beamline

The critical beamline components relevant to the energy resolution are the undulator source, the PGM and the exit slit. M1 and M2 are horizontally deflecting optics with plane surfaces in the sagittal direction resulting in negligible contributions to the energy resolution. The PGM (manufactured by BesTec GmbH) follows the classical SX-700 design very similar to that implemented at the ID32 at ESRF (Brookes *et al.*, 2018 ▸). The double-bouncing configuration ensures that monochromatic X-rays propagate parallel to the incoming beam. The variable line density of the gratings follows a polynomial equation, making monochromatic X-rays converge and focus at the exit slit. The vertical exit slit usually operates between 10 and 50 µm for selecting certain energy bandwidth.

For the plane VPG gratings (*i.e.* the radius of curvature *R* ≃ ∞), the VLS coefficients are optimized by zeroing the grating equation (4), the *f*_20_ (5), *f*_30_ (6) and *f*_40_ (7) terms at one reference energy (Appendix *A* [App appa]). As both the grating and the focus equation are fulfilled simultaneously across the whole energy range, the beamline works at a fixed *c*_ff_ value (cosβ/cosα) unlike the cPGM scheme where the *c*_ff_ value is a variable. Note that one could minimize the impact of the power-induced deformation on the M3 pre-mirror by optimizing *c*_ff_ (Reininger *et al.*, 2008 ▸). We discuss the effect of the absorbed power further in Section 2.5.

Contributions to the beamline energy resolution from the photon source, the PGM optics, and the exit slit are expressed in Appendix *B* [App appb]. The total energy resolution of the beamline is a vector sum of the four contributions.

**Figure 2:**
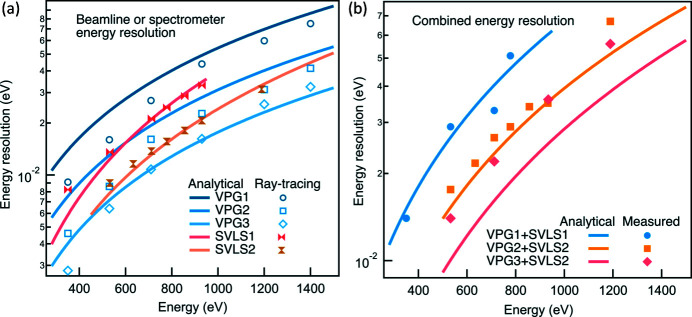
I21’s energy resolution. (*a*) The analytical and ray-traced energy resolution of the beamline and the RIXS spectrometer. (*b*) The analytical combined energy resolution with the experimental data.

It is noticeable that the longer the beamline entrance arm *r*_1_ or the exit arm *r*_2_ (at a fixed exit slit opening), the smaller the contributions from the source size and the exit slit. To achieve the best possible energy resolution, *r*_1_ and *r*_2_ are optimized to 43 m and 28 m, respectively, by considering the space constraints of the internal beamline and the external spectrometer hall. We highlight that the PGM optic slope errors (δ_Gr_ and δ_PM_) should reflect the final surface quality including the surface deformation induced by the mechanical clamping, and the angular instabilities caused by cooling water and the PGM mechanics. For the design of the I21 beamline, we paid significant effort to minimize the effective PGM optics surface slope errors. At the very early stage of the beamline construction, we purchased VPG grating substrates with specifications of 0.1 µrad slope error [the root mean square (RMS) value is used throughout unless otherwise stated]. For the optimization of the mechanical clamping, a comprehensive metrology study was performed in the Optics Metrology Lab at Diamond using the Diamond-NOM slope profiler (Alcock *et al.*, 2016 ▸; Nistea *et al.*, 2019 ▸). This study found that the surface slope error is remarkably sensitive to the mechanical configuration and the procedure of how the cooling mechanics are clamped onto the gratings. Using metrology feedback, the design and clamping procedure were optimized to minimize the strain induced in the optics. One needs to be meticulous in future designs if a surface slope error of <0.05 µrad is desired. For the M3 pre-mirror, because of the high absorbed power, we chose an internally water-cooled mirror design. The final achievements are slightly better than the design specifications of 0.2 µrad for all PGM optics. Table 4 ▸ summarizes the VPG grating parameters including the optimized surface slope errors. We show in Fig. 2 ▸(*a*) the analytical and ray-traced beamline full width at half-maximum (FWHM) energy resolutions based on the measured slope errors. All ray-tracing results presented here were performed using *SHADOW* (Sanchez del Rio *et al.*, 2011 ▸).

The I21 beamline is equipped with a gas cell as a standard component for quantifying the beamline energy resolution. It was used at the early stage of the beamline commissioning before the RIXS spectrometer became online. Most of the commissioning was performed at the Ne *K*-edge (∼868 eV). The energy resolution extracted from the deconvolution analysis matches well with the design; however, it starts to plateau at <50 meV due to the fitting uncertainty.

### 2.3. The energy resolution of the secondary spectrometer

From the optical scheme point of view, the I21 RIXS spectrometer is equivalent to a SVLS grating beamline. This parallel is stressed in the I21 spectrometer grating mechanics being labelled as the spherical grating monochromator (SGM). Given the space constraints of the spectrometer hall, we optimized the nominal length of the spectrometer at the reference energy to 13 m. Considering the limited translation ranges of the detector stage, we designed two SVLS gratings to cover the core energy range of 280–1500 eV. A third SVLS grating covers the intermediate X-ray energy range of 2–3 keV. The SVLS grating line density (*a*_0_ coefficient) and the nominal incident angle were optimized for the sake of the best energy resolution and the maximal vertical acceptance angle (for more details see Section 3.3). The higher-order VLS coefficients and the radius of curvature of the SVLS substrates were optimized by zeroing equations (4), (5), (6) and (7) shown in Appendix *A* [App appa]. We skip the discussion of the optimization procedure which is consistent with that developed by Strocov *et al*. (2011 ▸).

Concerning contributions to the spectrometer energy resolution, the vertical focal beam at the sample position, the SVLS grating optics and the CCD area detector represents the photon source, the diffraction optics and the exit slit, respectively. The detailed forms of these contributions are similar to equations (9), (10) and (12).

**Figure 3:**
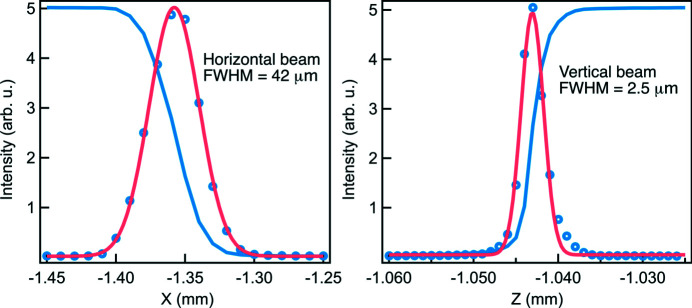
The measured focal beam size at the sample position with VPG2 at 1 keV and the exit slit opening at 10 µm FWHM. The blue open circles are the first derivative of the blue curve from a knife-edge scan, and the pink curve is the Gaussian fit.

Fig. 3 ▸ shows the focal beam size at the sample position with a fixed exit slit opening of 10.0 µm FWHM using the VPG2 grating at 1 keV. The ray-tracing vertical focal beam size at the sample is about 2.0 µm FWHM. The actual measured value is ∼2.0 µm, 2.5 µm and 3.0 µm FWHM at around 1 keV for VPG1, VPG2 and VPG3, respectively. The actual vertical beam size for VPG1 is consistent with the ray-tracing result; however, it becomes greater for VPG2 and VPG3. The horizontal focal beam size is ∼40 µm FWHM independent of the beamline gratings, consistent with the ray-tracing result. The slightly worse vertical focus at the sample is likely due to the beam footprint extending beyond the size of the polished area in the vertical plane. The M4 mirror is very curved in this direction, so it becomes increasingly difficult for mirror manufacturers to polish the optical surface as one moves away from the centre of the optical surface. The operational nominal *c*_ff_ for VPG1, VPG2 and VPG3 is 2.0, 3.0 and 5.0, respectively. As a consequence, the vertical footprint on the M4 mirror becomes larger from VPG1 to VPG3, hence gradually greater vertical focal beam size at the sample position.

Similar to the VPG gratings, we specified an effective slope error of 0.2 µrad for the SVLS gratings whose curvature is only along the tangential direction, *i.e.* retaining a cylindrical shape. Although a slope error of 0.05 µrad is achieved for the substrates, the finally optimized slope error is around 0.15 µrad after the grating clamping. Note that the measurement has slightly larger error bars than that of the VPG gratings due to the curvature and the transparency of the silica substrates. Nevertheless, we reiterate that the mechanical clamping is pivotal to the effective slope error. The measured vibrational stability of the SGM mechanics is ∼10 nrad, which is negligible for the current setup. All the SVLS grating parameters are shown in Table 5 ▸.

For the CCD detector, only commercial products were assumed to be available for the early beamline operation. Due to the charge spread-out in the soft X-ray energy range, the effective spatial resolution of CCD detectors is about 24 µm FWHM greater than the typical pixel size of 13.5 µm (Ghiringhelli *et al.*, 2006 ▸). For the SVLS spectrometer, the X-ray focal plane can be designed to a grazing-incidence angle (γ) so as to minimize the contribution from the CCD detector. For I21, the operational γ angle of the detector is optimized to be between 10° and 40° for the energy range of 280–1500 eV. The automation of the detector angular motion is achieved via a ferrofluidic seal design. For each energy position, although the detector is supposed to work at its optimized geometry, we notice that the energy resolution within the detector (one inch square) is tolerable within the operational γ angle.

The energy resolution of the spectrometer is a vector sum of the above three contributions. For the energy position other than the reference energy, the grating equation (4), the focus equation (5) and the coma equation (6) are fulfilled simultaneously. The horizontal (vertical) translation range of the detector is 5200 (1400) mm to cover the core energy range 280–1500 eV. Based on the actual achieved SVLS grating slope error (0.15 µrad) and the actual averaged vertical beam size (2.5 µm), we performed the analytical calculation of the spectrometer resolution together with the ray-tracing results, shown in Fig. 2 ▸(*a*). Note that the originally specified energy resolution of the spectrometer, based on slightly different source (2.0 µm) and grating slope errors (0.2 µrad), is comparable with the current calculations.

### 2.4. The total combined energy resolution

To obtain the total combined energy resolution, we take the vector sum of the beamline and the spectrometer FWHM energy resolutions and present the analytical result in Fig. 2 ▸(*b*). In addition, we plot the actual achieved energy resolution via measuring the elastic peak line width at the CCD detector for various grating combinations. The measured energy resolutions of the VPG1 + SVLS1 and VPG2 + SVLS2 match very well with the design throughout the whole energy range. However, for the VPG3 + SVLS2, the reality is always slightly worse than the theory. As explained, this may be partially due to the slightly larger vertical focal beam size (3.0 µm FWHM). We highlight that achieving the optimized energy resolution is straightforward owing to a free parameter available in the parameter set of the spectrometer.

**Figure 4:**
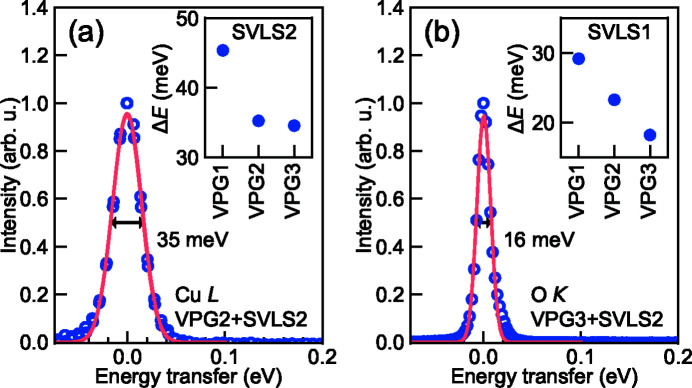
Elastically scattered X-ray beam at (*a*) Cu *L*- and (*b*) O *K*-edges from a carbon tape, fitted with Gaussian line-shapes of FWHM 35 and 16 meV, respectively. Insets show the achievable energy resolution at I21 for the three PGM gratings VPG1 (600 l/mm), VPG2 (1000 l/mm) and VPG3 (2000 l/mm) in combination with SGM gratings SVLS1 (1500 l/mm) and SVLS2 (2700 l/mm) at Cu *L*- and O *K*-edges.

**Figure 5:**
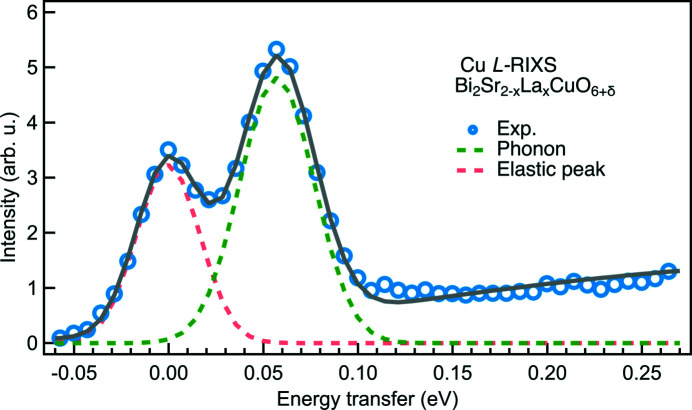
Bi_2_Sr_2–*x*_La_*x*_CuO_6+δ_ (*x* = 0.6) Cu *L*-edge RIXS spectrum at *q*_∥_ = 0.635 Å^−1^ projected along the crystalline *a*-axis acquired for 10 min using the grating combination of VPG2 and SVLS2 with an energy resolution of 38 meV (Li *et al.*, 2020 ▸). The pink dashed line is the elastic peak and the green dashed line is the bond-stretching phonon. The black solid line is a cumulative fit of the components and a linear background from paramagnons.

Fig. 4 ▸ displays the elastic peaks measured at the Cu *L*_3_-edge (930 eV) and the O *K*-edge (532 eV) using VPG2 + SVLS2 and VPG3 + SVLS2 grating configuration, respectively. The elastic peaks are fitted by Gaussian functions although there are indications of very small aberrations from the beamline optics. To demonstrate the RIXS data quality, we present a typical Cu *L*_3_-edge RIXS spectrum from a Bi_2_Sr_2–*x*_La_*x*_CuO_6+δ_ cuprate superconductor sample in Fig. 5 ▸. The high energy resolution of ∼38 meV and high photon throughput enable us to acquire a good statistical RIXS spectrum in 12 min where the bond-stretching phonons are clearly resolved from the elastic peak.

### 2.5. Other contributions to the energy resolution

Heat load on the optics is inherently associated with high-flux X-ray sources due to their high power. The M1 and M2 mirrors, which were designed and built by IDT Ltd, are directly cooled by a gallium–indium eutectic filled into a notch on the top surface of the optics. The eutectic itself is cooled by water through a metal fin. This cooling concept delivers sufficient cooling efficiency and, importantly, it avoids the mechanical clamping and minimizes cooling-induced vibrations. The most detrimental effect of the absorbed power on the energy resolution is the local surface deformations of the PGM optics along the tangential direction. A heat bump over the beam footprint alters the radius of curvature of the optics. To mitigate the impact, the M3 mirror is internally water-cooled through narrow channels just 1 mm beneath the mirror surface. The VPG gratings are also water-cooled through copper pipelines attached to the side of the gratings. The rest of the optics and mechanics downstream of the PGM are uncooled. In the dVLS scheme, the altered focal condition of M3 due to the absorbed power can be partially mitigated by modifying the operational *c*_ff_ value. We have observed that the *c*_ff_ value corresponding to the best energy resolution changes as a function of the cooling-water flow rate. In other words, the *c*_ff_ value correction works, though the non-spherical curvature may remain as part of the energy resolution broadening. The latter could be another potential source of detrimental contribution to the VPG3 energy resolution.

In the first year of the beamline operation, we experienced a gradual deterioration of the energy resolution. For instance, at the Cu *L*_3_-edge, the energy resolution increased monotonically from ∼35 meV to ∼55 meV. The resolution at the rest of the other energy positions also decayed similarly. Surprisingly, inhomogeneous energy resolution was found as a function of the M3 mirror’s transverse position. Visual inspection revealed that serious carbon contamination has developed near the interface of the cooled and the uncooled area. This was most likely due to the accidental beam heating from the initial X-ray commissioning during which the X-ray beam was placed close to the edge of the cooled zone. Subsequent optical metrology showed that the mirror surface had deformed significantly with the worst area having a slope error of 50 µrad. The deterioration of the energy resolution is correlated to the surface deformation induced by the beam heating. Note that there was no obvious change in the vacuum pressure in the PGM over the course of the first year of operation. Replacement of the damaged M3 mirror by an identical one has restored the energy resolution, and it has remained stable after we constrained the X-ray beam far away from the uncooled region.

Coma and spherical aberrations are detrimental to microfocusing using grazing-incidence X-ray optics. For instance, we revealed that the beamline energy resolution worsens if more than 4σ of the vertical divergent beam (σ is the RMS value of the vertical divergence) is accepted at the entrance of the PGM. Here the coma aberration of the PGM optics becomes non-negligible under the large vertical beam illumination. A similar situation happens for the spectrometer: the energy resolution deteriorates if the vertical acceptance of the SVLS grating is greater than 3 mrad, potentially due to non-negligible coma and spherical aberrations.

In reality, the vibration instability is another source of contribution to the energy resolution. The PGM and SGM internal mechanics are isolated from their external chambers which are supported by granite blocks. The exit slit, M4 mirror mechanics and the sample chamber also rest on granite supports. For the spectrometer, the SGM mechanics is built on top of granite air-bearing systems. The entire spectrometer steel structure is an in-house design which achieved the vertical (horizontal) vibration instability of ∼50 nm (100 nm) up to 100 Hz near the detector at a height of 2.0 m above the floor (Howell *et al.*, 2018 ▸). To ensure the same floor stability between the whole beamline and the spectrometer, the I21 spectrometer hall floor is piled using the same pile spacing and length as the Diamond internal floor. The pattern of piles was optimized to maximize the floor stability at the exit slits, sample position and radial pattern for the spectrometer air-bearing systems. The piled floor has achieved a vibration stability of ∼20 nm RMS between 1 and 100 Hz. Note that the vibrational stability does not impact much on the current performance. However, it may contribute in the future once the quality of the grating optics are improved.

## 3. X-ray photon throughput

### 3.1. A long straight and a long undulator

RIXS is a very photon hungry technique. For a high-resolution RIXS instrument, the number of photons accumulated at the detector is even more scarce due to the largely reduced energy bandwidth and the collection angle of the spectrometer.

**Figure 6:**
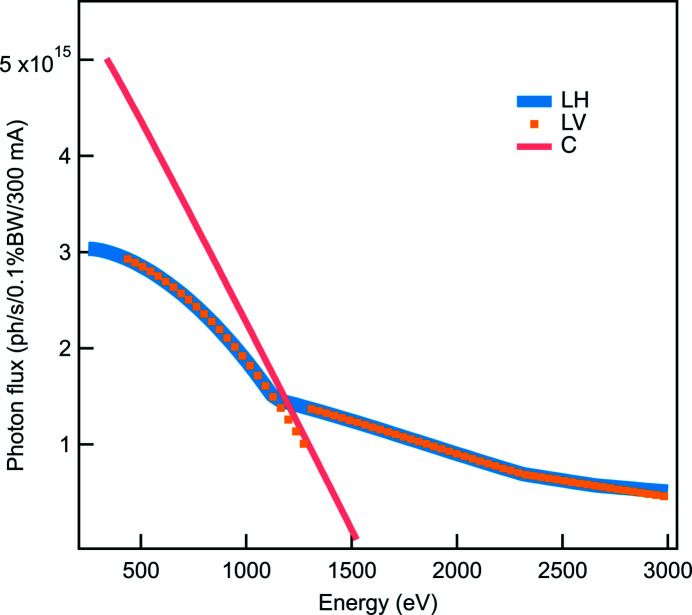
Simulated photon flux transmitted through the I21 front-end as a function of energy for three polarizations: linear horizontal (LH), linear vertical (LV) and circular (C). The simulation was done for the existing front-end aperture, which has an angular acceptance of 178 µrad horizontally and 143 µrad vertically. This allows >99% of the beam to pass through at the minimum energy of 250 eV. Circularly polarized light is available up to 1500 eV using the first harmonic. For linearly polarized light, the highest flux is provided by the first harmonic up to around 1200 eV, while the third harmonic provides the highest flux up to 2300 eV. Higher harmonics (up to the 11th harmonic) provide the maximum flux for linearly polarized light up to 3000 eV.

When the I21 beamline was proposed, a long straight section was selected so that a long undulator could be installed to provide a high photon flux. Eventually, a single 5 m-long helical undulator was designed and built by the in-house insertion device group at Diamond Light Source. The photon flux was simulated using the *Spectra* code (Tanaka, 2021 ▸) and is presented in Fig. 6 ▸. Note that all photon fluxes presented here, both calculated or measured, are for a ring current of 300 mA. Note that the high energy range (2–3 keV) can only be achieved by higher-order harmonics of the undulator in linearly polarized mode. The final choice of the period is based on the requirement of the minimum energy of the beamline.

### 3.2. Optimizing mirror reflectivities

**Figure 7:**
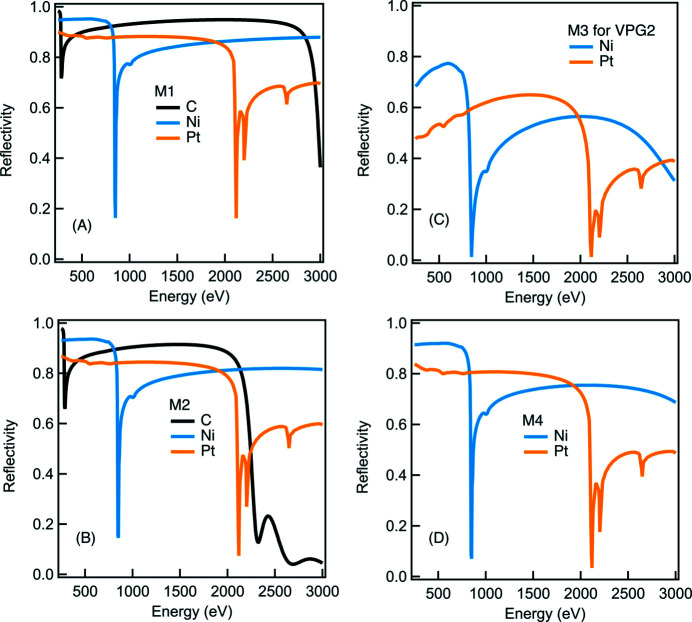
The simulated reflectivity of M1, M2, M3 and M4 mirrors as a function of energy. Note that the reflectivity of the M3 mirror is based on its corresponding α angle for each energy position.

**Figure 8:**
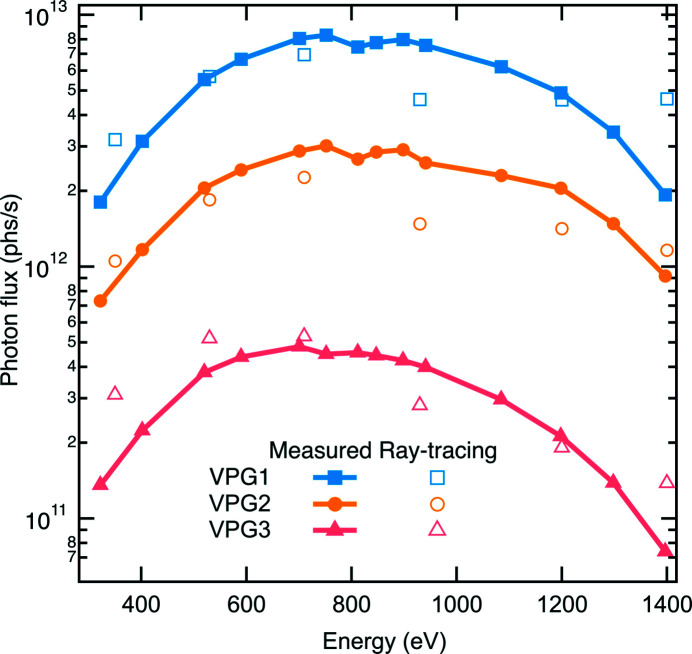
The measured (filled) and ray-tracing (open) monochromatic photon flux as a function of energy with the exit slit opening of 10 µm at a ring current of 300 mA.

We chose the dVLS optics scheme for achieving a high energy resolution but also for attaining the minimized number of optics compared with other optics schemes. All mirror optics were optimized to obtain a high reflectivity. For instance, M1, M2 and M4 mirrors work at shallowest possible grazing-incidence angles to maximize the photon throughput of the beamline. Additionally, multiple coating stripes of Pt, Ni and amorphous carbon were applied to both M1 and M2 to yield a high reflectivity throughout the entire energy range [see Figs. 7 ▸(*a*) and 7(*b*)]. For the strongly curved M4 ellipsoidal mirror, two identical optics (with a single layer of Pt and Ni coating stripe on each) are vertically staggered to cover the whole energy range [see Fig. 7 ▸(*d*)]. The same coating strategy (Pt and Ni) was applied to the M3 pre-mirror which works very effectively as the Ni coating yields about 50% higher reflectivity than the Pt coating for a photon energy lower than the Ni *L*-edges and above 2 keV [see Fig. 7 ▸(*c*)]. In Fig. 8 ▸, we show the monochromatic photon flux measured at a ring current of 300 mA using a calibrated photodiode AXUV-100 installed downstream of the exit slit with an opening of 10 µm FWHM. The ray-tracing results are also presented in Fig. 8 ▸.

**Figure 9:**
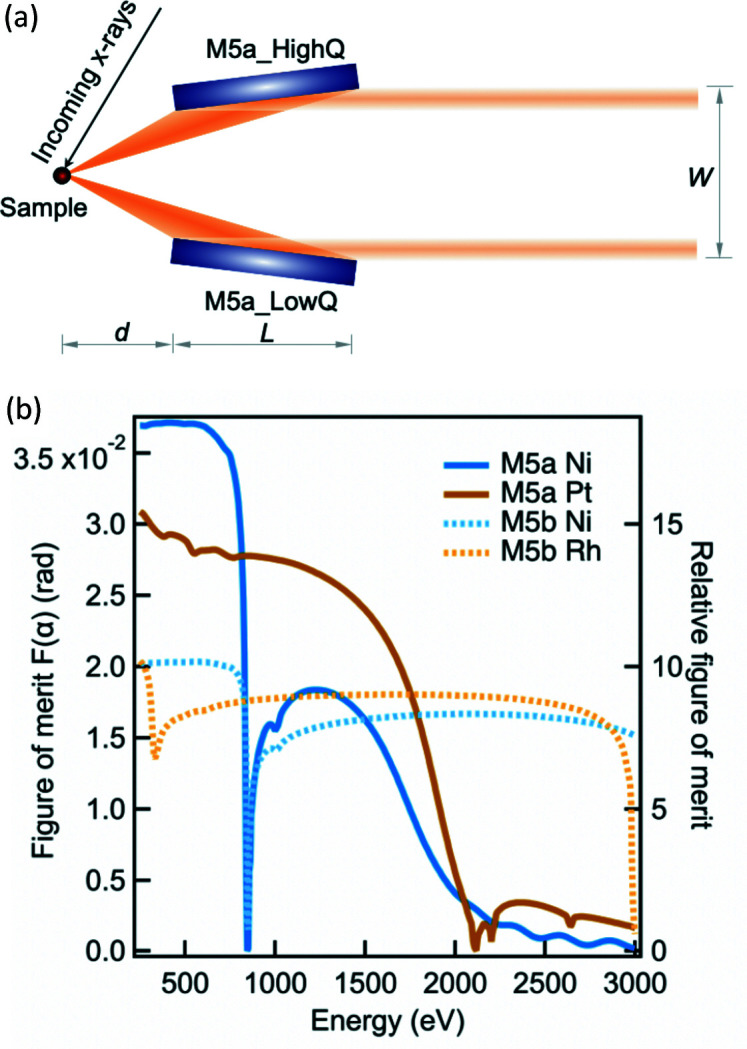
(*a*) Schematic top view of the M5a optics layout. *d*, *L* and *W* are equal to 55 mm, 190 mm and 30 mm, respectively, as optimized for the I21 beamline. (*b*) The simulated figure of merit of both M5a and M5b mirrors as a function of energy.

X-ray photon throughput of the RIXS spectrometer would have been significantly compromised if we simply adopted the conventional divergent SVLS optics scheme. The size limitation of the detector poses severe constraints on the collection angle of a long spectrometer. We investigated the collection mirror systems to enhance the horizontal angular acceptance Ω_H_. Eventually, we implemented two plane-paraboloidal mirrors (M5), symmetrically placed as close as possible to the sample so as to maximize Ω_H_. M5 are sagittally placed in the beam path with a fixed geometry having the plane (paraboloidal) surface in the sagittal (tangential) direction. In other words, M5 does not contribute to the energy resolution but only collimates X-ray photons in the horizontal plane [see the top view of the M5 schematics in Fig. 9 ▸(*a*)]. The optimization of the grazing-incidence angle α is based on a figure of merit *F*(α) which is the effective collection angle, *i.e.* approximates to the product of the mirror reflectivity *R*(α) (based on common coating materials of Pt or Ni) and the collection angle Ω_H_(α), 

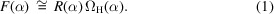

For a mirror operating in a grazing geometry, the collection angle Ω_H_(α) can be approximately expressed as

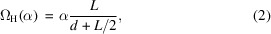

in which *d* is the distance between the sample and the close end of the mirror (*d* has a minimized value of 55 mm due to the space constraint), *L* stands for the mirror length which is dependent on the incidence angle α and must fulfil that the total horizontal span of the two stripes of beam is not greater than the ruled grating width (*W* = 30 mm),

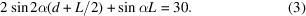

**Figure 10:**
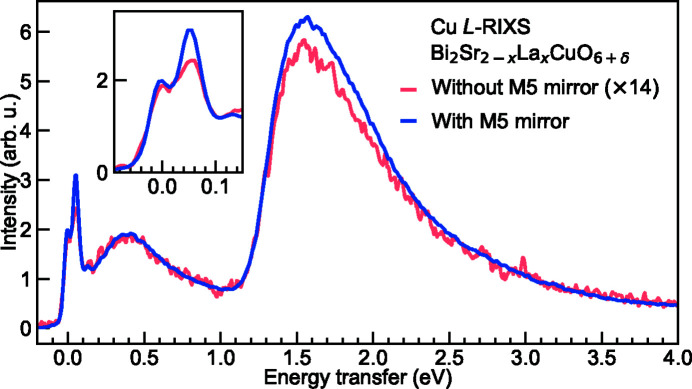
Bi_2_Sr_2–*x*_La_*x*_CuO_6+δ_ (*x* = 0.2) Cu *L*-edge RIXS spectra collected with and without M5 collecting mirrors and normalized to the incident X-ray flux. The inset shows the zoom into the low-energy excitations of the spectra.

In addition we set the criteria that the figure of merit *F*(α) has to maintain a relatively high value up to the energy of around 1500 eV. Eventually we optimized α and *L* to be 2° and 190 mm, respectively. For instance at the reference energy of 930 eV using SVLS2, this corresponds to an effective horizontal collection angle of 27.6 mrad in contrast to ∼2 mrad without the M5 mirror. For the 2–3 keV high-energy range, we applied the same optimization method and found that the α angle is optimized to 1° based on a metal coating of Ni or Rh. As a result, two pairs of M5 mirrors are implemented for the M5 system (details of the mechanical setup are discussed in Section 4). Similar to the beamline mirrors, we also applied multiple coating stripes to M5 for maximizing the reflectivities benefiting from the vertical plane shape. In Fig. 9 ▸(*b*), we present the absolute figure of merit and the relative figure of merit for both the core-energy (M5a) and the high-energy (M5b) mirrors. Note that the relative figure of merit demonstrates the gain of the horizontal acceptance after implementing M5 comparing with a fixed horizontal acceptance angle of 2 mrad without the M5 mirror. The latter is fixed to the value at the reference energy of 930 eV which in principle is a variable dependent on the SVLS grating and the sample-to-detector distance. In Fig. 10 ▸ we show Bi_2_Sr_2–*x*_La_*x*_CuO_6+δ_ Cu *L*-edge RIXS spectra collected with and without one of the M5a mirrors. The RIXS spectral line shape is consistent between the two, despite a factor of 14 difference in their intensities. The actual gain of the throughput matches well with the analytical calculation.

The substantial gain of the photon throughput is not without a price. M5 mirrors have a considerable magnification ratio of ∼100 requiring very delicate alignment with high precision and reproducibility. Measuring RIXS signals throughout the momentum space is a critical requirement for most single-crystal RIXS studies; therefore the X-ray focal beam has to meet both the pivot point of the M5 mirrors, which is the true 2θ rotation centre, and the pivot point of the manipulator rotation stage which is the true θ rotation centre, within 20 µm over a large angular range of 150°. More details of the mechanical setup are explained in Section 4. Besides the major demand in optics alignment, the close proximity (< 5 mm) between the M5 mirror system and the sample manipulator requires diligent mechanical designs for the sample manipulator and the sample transfer systems.

The application of a single M5 mirror has been a default operational mode for most users. Operating with two M5 mirrors doubles the RIXS efficiency for samples having no momentum dependence. For single-crystal materials, adding the second M5 mirror makes the alignment more complex, particularly due to the limited size of the current CCD sensor.

One minor point of using collecting optics is the slightly compromised momentum resolution. It increases from ±4 × 10^−4^ (2.3 × 10^−4^) to ±0.01 (0.006) Å^−1^ at the Cu *L*_3_- (O *K*-) edge. Although this is more than a one order of magnitude increase in the momentum resolution, it is still sufficient for most momentum-resolved RIXS studies.

### 3.3. Optimizing grating efficiencies

**Figure 11:**
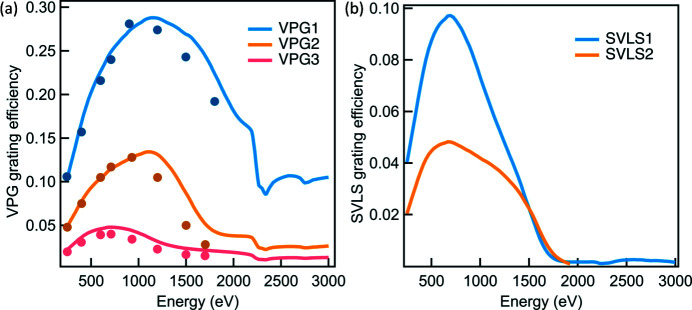
The simulated VPG and SVLS grating efficiencies (lines) as a function of energy. The results of the at-wavelength measurement (filled dots) on VPG gratings are also provided.

One of the major constraints of the photon throughput of soft X-ray beamlines is the grating efficiency. To achieve a high energy resolving power (*e.g.* >20000), the grating efficiency is typically below 10%. For the I21 beamline, we designed and optimized three VPG gratings with line densities of 600 lines mm^−1^ (l/mm), 1000 l/mm and 2000 l/mm, to cover the core energy range (280–1500 eV), for the high-flux, medium-resolution and high-resolution mode, respectively. Fig. 11 ▸(*a*) displays the simulated grating efficiencies together with the corresponding at-wavelength measurements performed at the Optics Beamline at BESSY (Eggenstein *et al.*, 2013 ▸). The *REFLEC* program (Schäfers & Krumrey, 1996 ▸) was used to simulate the grating efficiencies. Note that each grating works at a more or less fixed *c*_ff_ value across the vast majority of the energy range. The energy range of 2–3 keV is covered by the VPG4 grating which falls out of the main focus of the paper.

Optimizing the efficiency of the SVLS gratings is more complex than that of the VPG gratings. The primary drive is to achieve the targeted energy resolution with highest possible effective vertical collection angle which is the product of the grating efficiency and the vertical collection angle Ω_V_. Due to the large energy range and the limited vertical and horizontal translation ranges of the detector platform, two SVLS gratings are needed to cover the energy range of 280–1500 eV. We specified an energy resolution of ∼15 meV (25 meV) at the reference energy of 530 eV (930 eV) for the SVLS1 (SVLS2) grating, based on the following parameter set: the spectrometer length (at the reference energy) = 13000 mm, SVLS slope errors = 0.2 µrad (RMS), length of SVLS gratings = 200 mm, source size = 2.0 µm (FWHM), CCD resolution = 24 µm (FWHM) with γ angle = 20°. In order to achieve this, the analysis delivers a grating line-density of 1500 l/mm (2700 l/mm). For both SVLS gratings, a common nominal grazing-incidence angle of 2.4° was chosen to fulfil the designed translation ranges of the detector. Also, an optimized grating entrance arm *r*_1_ of 2200 mm is chosen for the reference energy so that the range of *r*_1_ over the entire energy range fits within the mechanical constraints of the SGM. Full optimization of the SVLS gratings was made by using our own analytical code, the *REFLEC* program as well as the *TraceVLS* program from Strocov *et* *al*. (2011 ▸). We show in Fig. 11 ▸(*b*) the simulated efficiency of each SVLS grating.

### 3.4. Carbon contamination

Carbon contamination is a serious issue for soft X-ray beamlines. A thin amorphous carbon layer normally builds up on optics surfaces and deteriorates its reflectivity. At the I21 beamline, we set a stringent vacuum pressure threshold for the initial beamline commissioning. All optics components and chambers are always baked up to around 110°C for vacuum conditioning after every vacuum intervention. During operation, the pressure of the M1 and M2 systems is <1 × 10^−9^ mbar under the X-ray exposure. The pressure of the PGM system is <5 × 10^−10^ and <5 × 10^−9^ mbar without and with X-rays, respectively. Unlike other beamlines, where a low dose of O_2_ is leaked to the PGM system to actively minimize carbon contamination, we found that the optics reflectivity is well maintained after the first few years of user operation, and hence a partial O_2_ pressure is not used. We would like to note that the Ni coatings on M1, M2 and M3 may be at risk of oxidizing in a partial O_2_ pressure, so this was another factor in our decision.

## 4. The I21 sample station and the RIXS spectrometer

**Figure 12:**
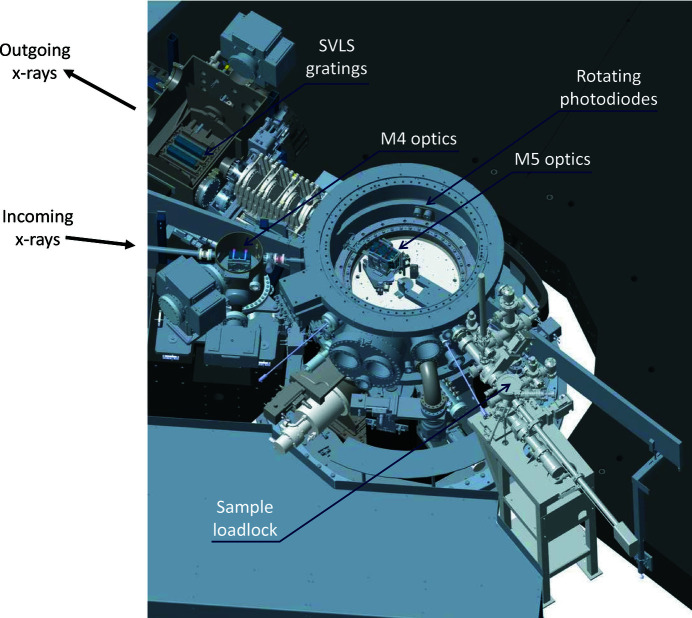
A rendering of the I21 sample station and the surrounding area.

One critical aspect of modern RIXS beamlines for the study of three-dimensional quantum materials is that one needs to be able to measure at an arbitrary point in reciprocal space. In practical terms, this means that one needs to be able to rotate the sample about at least three independent rotation axes that pass through the sample. It also means that one needs to be able to change the scattering angle: *i.e.* in the case of I21, one needs to be able to rotate the whole ∼15 m-long spectrometer arm. When one considers that all of the above also needs to be performed under ultra-high vacuum conditions (<1 × 10^−9^ mbar), and that the light emitted from the sample needs to be able to pass to the CCD detector located at the end of the spectrometer, then it is clear that this is a tremendous mechanical engineering challenge. Moreover, many quantum materials have novel behaviour at low temperatures (<50 K), so it is also important to be able to cryogenically cool samples. Finally, as described in Section 3.2, in order to enhance the SVLS spectrometer throughput it is vital to use horizontally collecting mirrors (M5) at the expense of broadening the momentum resolution in the horizontal plane. The effectiveness of these mirrors is enhanced by having them as close as possible to the sample position, and therefore at I21 these mirrors are installed inside the sample vessel. An overview of the sample area is presented in Fig. 12 ▸.

In this section, many of the mechanical and vacuum engineering aspects of the I21 sample station and the RIXS spectrometer are described in detail. We firstly describe the I21 sample manipulator. We then present the mechanics for the M5 mirror system, followed by a description of the sample vessel in which the manipulator and the M5 system are both installed. Finally, we describe the design of the RIXS spectrometer, and discuss the key design decisions that we made.

### 4.1. Sample manipulator

**Figure 13:**
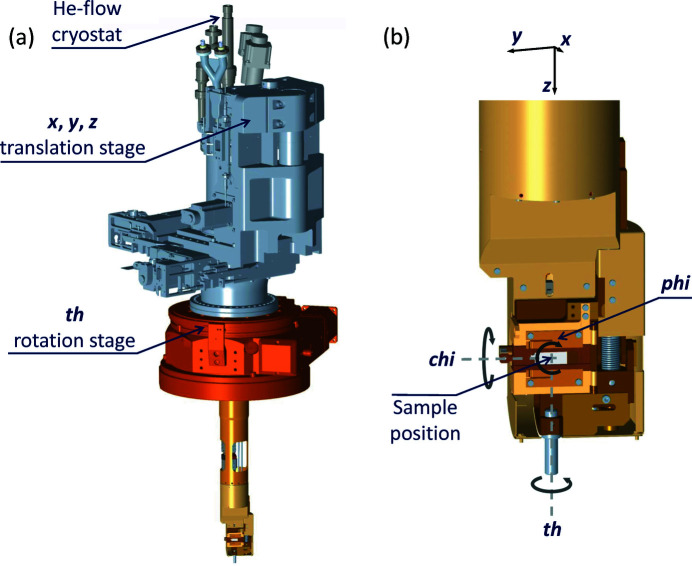
The I21 sample manipulator. In panel (*a*) the whole manipulator is shown, with the key out-of-vacuum components labelled. Panel (*b*) shows the sample holder of the manipulator, with all six degrees-of-freedom labelled.

The I21 sample manipulator is essentially a modified version of the sample manipulator in use at the I05 ARPES beamline at Diamond Light Source. Further details about the manipulator can be found in the I05 beamline paper (Hoesch *et al.*, 2017 ▸). Here we focus only on the modifications that were made for the I21 beamline. The manipulator is shown in Fig. 13 ▸.

One important modification was to enable drain current measurements to be performed on the sample, so total electron yield X-ray absorption measurements become applicable. This was accomplished by introducing a sapphire layer behind the sample puck holder, which at low temperatures is an excellent thermal conductor but a poor electrical conductor. This electrically isolates the sample so that it is possible to measure a drain current from it. The change also raises the lowest possible sample temperature achievable, so whereas at I05 <7 K is achieved, at I21 a lowest temperature of 9.5 K is obtained. However, this is still much lower than ∼20 K that most other RIXS beamlines can achieve at present.

The I05 manipulator design blocks incoming or outgoing X-rays with an angle ∼20°. While this is not a significant issue for most ARPES studies, this design would exclude certain grazing geometries which can be very important in momentum-resolved soft X-ray RIXS studies. Therefore, for the I21 manipulator, the sample position was shifted in the positive *x* direction (see Fig. 13 ▸) in order to enable grazing incoming/outgoing X-ray beams. Consequently the centre of rotation of the chi (χ) axis is displaced with respect to the centre of rotation of the th (θ) axis. Therefore in order to rotate χ, one needs to correct the sample position in both *x* and *z* as otherwise the sample would precess out of the beam. This precession correction has been implemented in software and is used routinely by users.

Another key difference relative to the I05 manipulator is the order in which the translation stages and the θ rotation stage are mounted out-of-vacuum. At I05 the rotation stage is mounted on top of the *XYZ* translation stage (so if the sample is moved in the horizontal *XY* plane, the θ rotation axis also moves), whereas at I21 the rotation stage is underneath the *XYZ* translation stage, and is fixed to the sample vessel. This means that at I21 the θ rotation axis is decoupled from the *XYZ* translation stage. This decision was made because it is critical for I21 that any part of the sample can be put on the θ rotation axis to eliminate sample precession. An alternative solution would have been to put the *XYZ* stage in vacuum, as was done at the ID32 RIXS beamline at the ESRF (Brookes *et al.*, 2018 ▸), but this would have been incompatible with the in-vacuum M5 mirror design and would also have compromised the base temperature achievable.

In addition, the I05 manipulator uses a differentially pumped rotary feedthrough (ZRP100) from VacGen as the θ rotation stage, but the eccentricity of such a stage is uncertain. Since any eccentricity of the rotation stage would manifest itself as a change in sample position as a function of θ, we chose to use a high-precision rotation stage (a HUBER Goniometer 420) which HUBER Diffraktionstechnik GmbH supplied with a differentially pumped rotary feedthrough integrated into the design. The eccentricity of this stage was found to be <5 µm.

**Figure 14:**
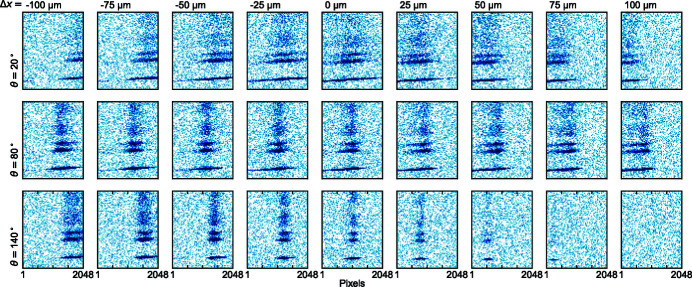
Panels showing the movement of the RIXS signal on an Andor CCD detector for different X-ray incident angles (θ) and sample *x* positions. The top, middle and bottom rows correspond to θ = 20°, 80° and 140°, respectively. The panels in each column correspond to increments of *x* in steps of 25 µm in negative or positive directions from the central position.

The alignment of the sample manipulator is critical for efficient operation of the I21 beamline. It is of paramount importance that the focal beam position is coincident with the sample manipulator θ rotation axis, otherwise the RIXS signal shifts from the centre of the detector when θ is changed. One can see this effect in Fig. 14 ▸, where the X-ray beam is deliberately shifted away from the θ rotation axis in 25 µm increments. Even after one 25 µm step, we already see that the beam is noticeably shifted from the centre of the detector screen when in grazing incidence (θ = 20°) or in grazing outgoing (θ = 140°) geometries. Such a high sensitivity is a direct consequence of the extreme magnification ratio of the M5 mirror optics. In practice we have found that the beam needs to be within 20 µm of the θ rotation axis in order to minimize such effects. These observations demonstrate that the low eccentricity of the HUBER rotation stage is absolutely vital to ensure smooth user operation.

As with the I05 design, the sample manipulator has the same angular range for the in-vac χ and phi (ϕ) axes. The detailed motion parameters are presented in Table 6 ▸. The I21 manipulator is compatible with standard Omicron pucks. Samples can be transferred via a loadlock which allows up to five samples to be installed at once.

### 4.2. The M5 mirror system

**Figure 15:**
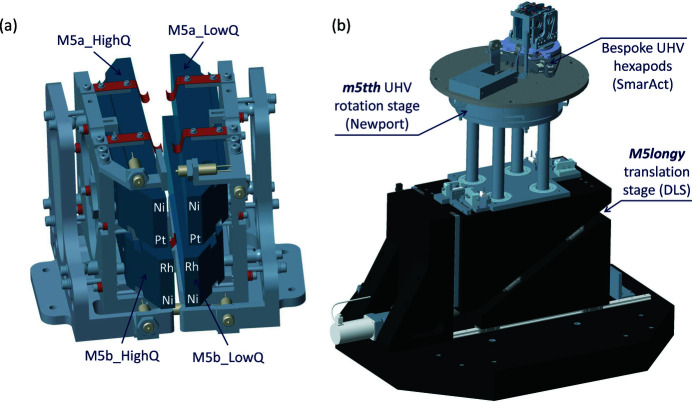
The I21 M5 collection mirror mechanics. Panel (*a*) is a rendering of the M5 optics and the mirror holders, while (*b*) shows the whole M5 mirror mechanics.

In Fig. 15 ▸(*a*) a rendering of the M5 optics in their mirror holders is shown. As described in the optical properties of M5 mirrors in Section 3.2, a pair of optics was implemented for obtaining the highest possible photon throughput in the core energy range up to 1.5 keV (M5a). An additional pair (M5b) was implemented for the high-energy range (2–3 keV). The mechanics were designed so that each of the vertical stack of mirrors could be clamped separately and any induced clamping distortions could be checked on the Diamond-NOM before installation (Alcock *et al.*, 2010 ▸, 2016 ▸). Each vertical stack of mirrors could then be installed into the main mechanics using three bolts from the back, minimizing the risk of any damage to the optical surfaces during installation.

The two vertical stacks of mirrors are mounted on two independent ultra-high-vacuum (UHV) compatible hexapods, manufactured by SmarAct GmbH. The two vertical stacks of mirrors are so close to each other that the two SmarPods are effectively nested together, but they were carefully designed so that it is not possible for them to clash with each other. As one can anticipate from Fig. 15 ▸(*a*), the close proximity of the mirror holders and the mirror optics means that, without mitigation, one mirror could easily collide into another using the independent hexapods. This problem is circumvented by the addition of seven in-vacuum limit switches mounted on the mirror holders, which alert users if the mirror mechanics are in a dangerous position. These limit switches also act as hard stops, making it impossible for the mirrors to contact one another.

In Fig. 15 ▸(*b*) a rendering of the whole M5 mirror mechanics (with all other components removed, including the sample chamber) is presented. In operation, the M5 optics need to be rotated about a vertical axis to match the scattering angle of the spectrometer. This is achieved by using a UHV-compatible rotation stage from Newport (an RV350). The eccentricity of this stage is <5 µm.

As the I21 M5 mirror system consists of two horizontal pairs of mirrors, one needs to be able to translate the mirrors vertically by up to 90 mm to switch between the top pair and the bottom pair. This is done ex-vacuum by a granite wedge stage that was designed in-house at Diamond Light Source using rails from Schneeberger. The vertical motion is then transferred into ultra-high vacuum using the four posts shown in Fig. 15 ▸(*b*) which pass through four bellows mounted on the bottom of the sample vessel.

For operation at multiple values of 2θ scattering angle formed between the beamline and the spectrometer arm, it is critical that the eccentricity of the Newport rotation stage is minimized, that the rotation axis of the Newport rotation stage is as close to vertical as possible, *and* that any parasitic horizontal translations or parasitic rotations of the M5 vertical stage are minimized. The motion of the M5 vertical stage was checked via two methods: the horizontal translations were checked using a laser tracker system which was sensitive to motions down to 50 µm, while the parasitic angles and orientation of the rotation axis were checked using an autocollimator setup to measure the change in angle of the stage as a function of the position of the M5 vertical stage. After fine adjustment of the support structure between the granite wedge stage and the Newport rotation stage, we found no parasitic horizontal translations (down to ∼50 µm), while the parasitic rotations were below ±40 µrad.

**Figure 16:**
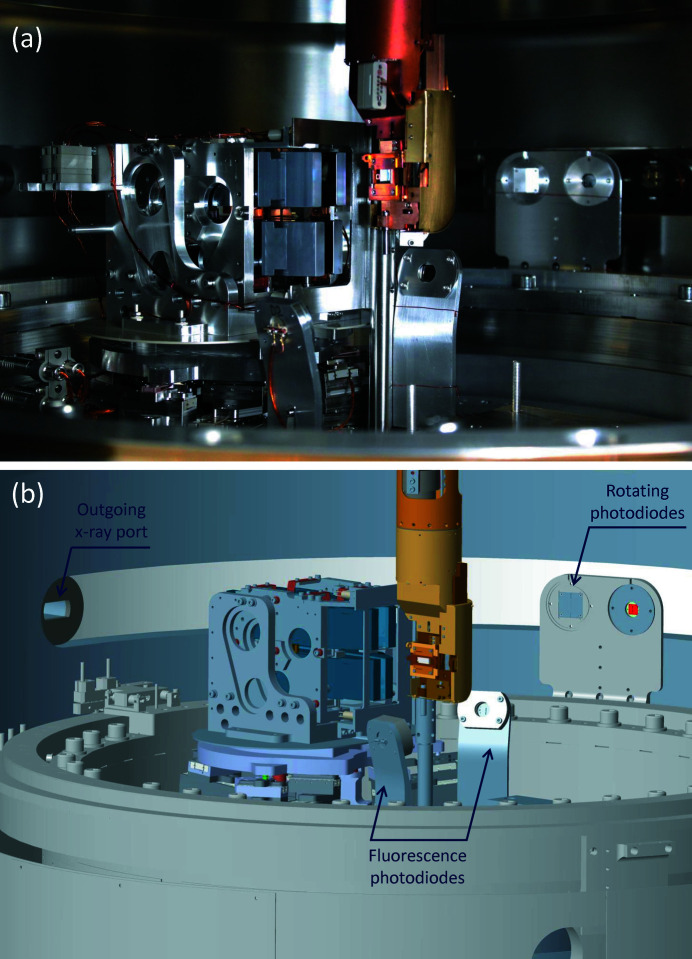
The in-vacuum components inside the I21 sample vessel. (*a*) A photograph through one of the large viewports on the sample vessel and (*b*) an engineering rendering of a similar view in the CAD model.

Fig. 16 ▸(*a*) shows a photograph of the inner part of the sample vessel which can be compared with a rendering of the 3D engineering model in Fig. 16 ▸(*b*). Besides the aforementioned optics and mechanics, two photodiodes are mounted on the Newport rotation stage close to the sample manipulator. These were initially anticipated to collect the X-ray fluorescence signal from samples for X-ray absorption spectra (XAS). Another set of photodiodes are installed on an in-vacuum slewing ring further away from the manipulator. When combined with the M5 mirrors, these rotary photodiodes are found to provide XAS data with a comparable quality to the fixed photodiodes. The rotary photodiodes also act as an important detector for the beamline alignment and the sample half-cutting alignment, which are crucial for setting up user experiments.

### 4.3. Sample vessel with continuously rotatable outgoing X-ray port

In order to be able to measure RIXS over a large range of scattering angles, one needs to be able to rotate an outgoing X-ray port on an ultra-high-vacuum sample vessel. One possible solution is to use very large differentially pumped rotary feedthroughs, but this is complicated by the fact that one needs to keep the incoming X-ray port fixed while rotating the outgoing X-ray port. Another solution is to use a triple-rotating flange, as is used at the SIX beamline at NSLS II (Dvorak *et al.*, 2016 ▸), which can use standard (if very large) differentially pumped feedthroughs but requires complicated motion control systems to make sure the port moves as required. We chose to use the solution pioneered by CINEL, which uses differentially pumped seals in a novel geometry, following the design used at the ID32 beamline at the ESRF (Brookes *et al.*, 2018 ▸).

The I21 sample vessel provides a large angular range (0–150°) for 2θ. This enables RIXS measurements to be performed at low scattering angles (<50°) which is essential for some science cases (Nag *et al.*, 2020 ▸). The base pressure achieved in the sliding-seal vessel is 2 × 10^−10^ mbar and is pumped with one 500 l s^−1^ ion pump (Gamma Vacuum 500T), a 300 l s^−1^ turbomolecular pump (Edwards Vacuum nEXT300D) and a cryopump (SHI Cryogenics Group Marathon CP-8). The sliding-seal vessel achieves a remarkable vacuum pressure of <7 × 10^−10^ mbar while the sliding seal is in motion thanks to the differential-pumping seal design from CINEL.

**Figure 17:**
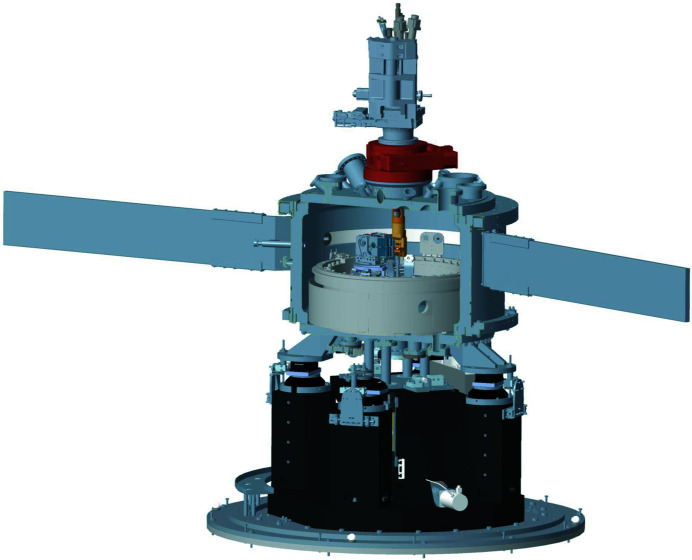
The I21 endstation. The sliding-seal sample vessel, the M5 mirror system and the sample manipulator are all shown.

To ensure the mechanical stability, the sample vessel is supported on four airpads resting on four granite posts around the M5 mirror system, as shown in Fig. 17 ▸. The initial alignment of the eccentricity between the manipulator rotation axis and the M5 rotation stage was done by using the airpads to move the whole sample vessel in the horizontal plane. Once the correct location of the sample vessel was found, the vessel was firmly secured in place.

A great effort was made in the early design of the sample chamber to ensure that there are two CF40 ports at exactly 90° to one another in the horizontal scattering plane. This was originally enabled to allow any sample precession to be monitored using the cameras, but in practice the extreme sensitivity of the M5 mirror system to the sample position (see Fig. 14 ▸) acts as a far more sensitive measure of the eccentricity of the sample. However the attention placed on ensuring excellent visibility inside such a complex sample vessel has proved to be worthwhile, as it helps make operation of the endstation more intuitive and straightforward.

### 4.4. The mechanical design of the RIXS spectrometer

**Figure 18:**
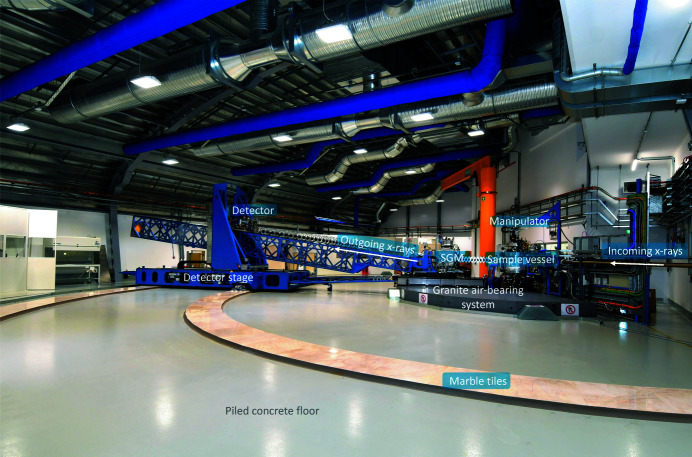
Overview of the I21 RIXS spectrometer.

An overview of the I21 RIXS spectrometer and the dedicated spectrometer hall are shown in Fig. 18 ▸. The optical design of the spectrometer was described in Section 2.1.

The SVLS gratings optics are accommodated in a SGM monochromator (BesTec) which follows a similar internal mechanical design as a typical PGM but with only one rotational axis. Because of the requirement of a variable entrance arm *r*_1_, the SGM mechanics is supported on an air-bearing system which works in combination with a rack and pinion drive system. Unlike the PGM, the SGM mechanics is equipped with two linear wedge stages underneath to accommodate the potential misalignment of the roll axis or the absolute height of the SGM. Since there is no absorbed power to be concerned with for the RIXS spectrometer, the SVLS gratings are only base-clamped without the need of cooling mechanics. The overall mechanical stability has been measured as ∼10 nrad up to 100 Hz.

The steel structure of the spectrometer was an in-house design by Diamond Light Source and manufactured by OCSAM. The horizontal (vertical) detector stage was made so as to cover a translation range of 5.5 m (2.0 m). The structure of the two stages were optimized to achieve the highest possible eigenfrequency of the first mode. The two motions are based on linear rails and equipped with absolute encoders. The lattice structure was designed to be light and stiff so as to support the long but retractable beam tube and bellows between the SGM and the detector vertical stage. The linking structure is flexible in the vertical direction but rigid in the horizontal in order to make the whole spectrometer acting effectively as one single mechanical component for the rotational motion of the scattering angle.

Similar to the ERIXS spectrometer design at ID32, the I21 spectrometer uses airpads to lift the detector platform when the spectrometer is rotated about the sample position. However, at I21 the SGM also moves on air-bearings, whereas at ID32 the system sits on a high-precision rail. In principle a rail could act as a spring, making the grating mechanics less stable. We chose to use an air-bearing so that when the air is switched off, the contact with the highly stable ground is maximized (see Section 5).

During the early stage of the user operation, we observed deformations near the interfaces of the marble tiles. These are troublesome for the air-bearing system which only lifts the entire spectrometer in the region of ∼40 µm. The heavy weight of the I21 spectrometer may be partially the cause of the deformations; however, the unavoidable settlement of the whole building may also be contributing to the issue. The situation is improving as we have performed repolishing of the granite and marble floors. We are also currently increasing the lifting capacity by making new air-bearing systems for the detector platform.

## 5. Beamline stability

We have demonstrated the importance of the high-frequency vibrational stability of optics and mechanics for achieving a good energy resolution. Equally, the long-term stability (∼hours) of the beamline components is also critical to ensure smooth RIXS data acquisition and in general user friendliness. At the I21 beamline, almost all motions are equipped with encoders operating in close-loop. For instance, for the vertical beam (relevant to the energy resolution), pitch angular motions of PGM and SGM optics are corroborated with highest possible angular stability in both the short term (vibrations) and the long term (drifts). We also designed a feedback system (up to 10 Hz) to control the horizontal beam position throughout the beamline. For the M1 and M2 mirrors, besides the coarse pitch angular motions supplied by the primary mirror mechanics (supplied by IDT Ltd), additional pitch motions of the individual optics are implemented via an in-vacuum piezo-drive mechanics. As part of the beamline diagnostics tools, drain current measurements were enabled from the blade edges of the second and third white-beam slits (WBS, supplied by BesTec) installed downstream of M1 and M2, respectively. Thus M1 fine pitch is auto-corrected by the differential drain current of WBS2 located 13.5 m downstream of M1. Similarly, M2 fine pitch is auto-corrected via the horizontally focused monochromatic beam imaged by a scintillator screen at the exit slit.

**Figure 19:**
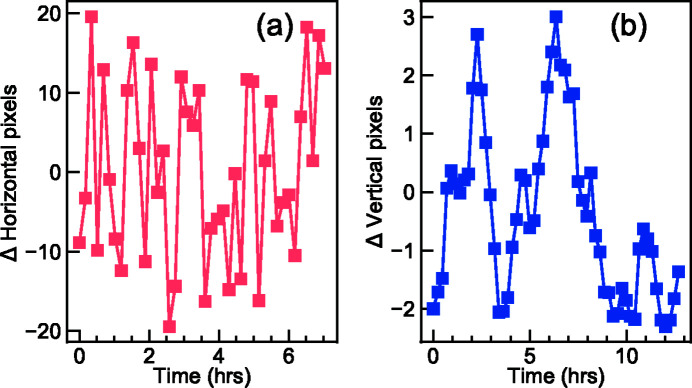
(*a*) Relative horizontal shifts in pixel values corresponding to the maxima of the RIXS signals on the CCD over time. (*b*) Relative vertical shifts in pixel values corresponding to the elastic peaks of the RIXS signals on the CCD over time.

In Fig. 19 ▸ we present a typical long-term drift of an elastic peak. Over the course of ∼7 h, the horizontal position of the elastic signal drifts within ±20 pixels during an energy detune RIXS measurement. This corresponds to a horizontal beam stability control of ∼±3 µm at the sample position. The vertical position of the elastic signal from a momentum-dependent RIXS measurement drifts within ∼±2 pixels which indicates that the combination of the SGM pitch angular stability and the detector stability is within ±80 nrad.

The environmental temperature stability of the whole spectrometer hall is closely controlled by the air-conditioning system to minimize the thermal drift of the beamline optics and the mechanics. For instance, the temperature stability of the spectrometer hall is controlled to be within ±0.1°C over the course of five days. The low thermal fluctuations of the spectrometer hall, together with the large steel structure which has minimal thermal expansion, ensure smooth user operations.

## 6. Beamline control systems, data acquisition and data analysis

The I21 beamline control system is realized using Linux and Windows servers interfaced to the equipment being controlled. These are connected across a network to workstations that provide the operator interface and other functionalities. The software for the control system is built on EPICS, which is an open source software platform used by many large-scale facilities.

For the data acquisition, we use the *Generic Data Acquisition* (*GDA*) system which is now implemented on almost all beamlines at Diamond. *GDA* provides a common Jython syntax scripting environment with graphics user interface and object customizations specific for the science performed at the beamlines concerned. For the I21 beamline, a specific perspective is designed for RIXS data acquisition in which common functionalities developed for other beamlines can be shared but one could also add specific requirements. All data collected at I21 are written in the NeXus file format (Könnecke *et al.*, 2015 ▸) in which rich metadata is captured including the positions of almost all critical beamline components and the values of various monitoring devices. This is, of course, highly beneficial for the downstream data analysis as well as for future reference.

RIXS data analysis software was developed to run within Diamond’s data visualization and processing package called *DAWN* (Basham *et al.*, 2015 ▸). Image background subtraction, data reduction from 2D images to 1D line spectra, and correlation between RIXS spectra were implemented using the *DAWN* data processing framework (Filik *et al.*, 2017 ▸). Also, *DAWN* was extended with the ability to aggregate line spectra into 2D or 3D datasets to enable plotting in energy and momentum spaces.

## 7. Conclusion and future outlook

We presented the optical design and the actual achievements of the I21 RIXS beamline facility at Diamond Light Source. The attained total energy resolution matches well with the theoretical value. Owing to the unique design of the collection mirrors, the I21 beamline features unprecedented X-ray photon throughput even at a very high energy resolution mode. We also elaborated on the detailed mechanical designs of the sample manipulator, the M5 collection mirror system, the sample vessel with continuously rotatable outgoing port, and the RIXS spectrometer. Practical lessons learned during the design, construction and optimization stages were introduced throughout the paper for interested readers.

The intermediate-energy RIXS (between 2 keV and 3 keV) will be soon available at the I21 beamline. The white-beam optics as well as the refocusing and collection mirrors were carefully designed and implemented in the construction phase. The only optics currently under commissioning are the corresponding VLS grating. In addition, we are also in a design phase for a full polarimeter analysis apparatus. In the future, the I21 beamline will be capable of disentangling the outgoing polarization of RIXS signal to aid the study of low-energy excitations.

We will constantly seek the improvement of the energy resolution and the photon throughput at the I21 beamline. Fundamentally the beamline energy resolution and the throughput are limited by the overall quality of the VPG gratings. New grating optics and improvements to their mechanics are required to achieve better performance. For the RIXS spectrometer, the energy resolution is mainly limited by the detector spatial resolution, the SVLS grating quality, and the vertical focal beam size. Significant efforts are needed in order to improve the total energy resolution in the future.

The I21 beamline has been recognized by the broad user community for being stable, reproducible and highly efficient. However, certain areas can still be improved. For instance, cooling or warming up the manipulator takes a significant amount of time which is sometimes detrimental to a short user experiment. The sample transfer system is complex, particularly due to the proximity of the manipulator and the collection mirrors. Finally we believe there is still potential to refine the automation of the beamline optimization, the data analysis and the remote control. This would not only help to ease the burden of increasing numbers of remote users but also allow to build up a more user friendly RIXS facility in the long term.

## Figures and Tables

**Table 1:** List of all mirrors up to the sample position Note that for M1, M2 and M3, any parameters with multiple values represent the measurement performed at different coating stripes. For mirror M4, they represent the measurement performed on the individual M4a or M4b mirror.

Optic	M1	M2	M3	M4a and M4b
Distance from source (m)	26.00	40.50	43.00	80.00
Shape	Plane	Tangential cylinder	Plane	Ellipsoid
Dimensions L × W × H (mm)	450 × 80 × 60	530 × 80 × 60	450 × 70 × 48	130 × 40 × 50
Angle of grazing incidence (°)	0.6	0.8	Variable	1
Coatings	C, Pt, Ni	C, Pt, Ni	Pt, Ni	Pt (M4a), Ni (M4b)
Tangential radius (km)	55.6 (C)	2.487 (C)	135 (Pt)	*A* = 5000 mm
	44.4 (Pt)	2.485 (Pt)	144 (Ni)	*B* = 52.4 mm
	45.8 (Ni)	2.490 (Ni)		*X*_*m*_ = 4000 mm
Sagittal radius (km)	42	21	12	See above
Tangential slope error (µrad, r.m.s.)	0.349 (C)	0.249 (C)	0.127 (Pt)	1.86 (M4a)
	0.316 (Pt)	0.235 (Pt)	0.154 (Ni)	1.94 (M4b)
	0.341 (Ni)	0.225 (Ni)		
Sagittal slope error (µrad, r.m.s.)	0.45	0.45	Not specified	2.8
Roughness (Å, r.m.s.)	1.73	2.00	2.82 (Pt)	3.0 (M4a)
			2.63 (Ni)	3.1 (M4b)
Manufacturer	Zeiss	Zeiss	InSync and JTEC	Zeiss

**Table 2:** Specification of M5 collecting mirrors after the sample position Note that the M5a (M5b) HiqhQ and LowQ mirrors’ slope errors are shown in the table.

Optic	M5a	M5b
Distance from source (mm)	150	150
Shape	Tangential parabola	Tangential parabola
Dimensions L × W × H (mm)	190 × 30 × 40	190 × 30 × 40
Angle of grazing incidence (°)	2	1
Coating	Ni, Pt	Rh, Ni
Tangential radius (km)	Vertex radius = 0.3654 mm	Vertex radius = 0.0914 mm
	Off-axis position = 149.817 mm	Off-axis position = 149.954 mm
Sagittal radius (km)	>10	>10
Tangential slope error (µrad, r.m.s.)	2.9 (HighQ), 3.4 (LowQ)	2.1 (HighQ), 1.9 (LowQ)
Sagittal slope error (µrad, r.m.s.)	4.8 (HighQ), 4.6 (LowQ)	3.1 (HighQ), 2.3 (LowQ)
Roughness (Å, r.m.s.)	2.53	1.96
Manufacturer	Zeiss	Zeiss

**Table 3:** Undulator parameters

Apple II helical undulator	HU56
Magnetic material	NdFeB
Maximum vertical magnetic field (T)	0.70
Maximum horizontal magnetic field (T)	0.43
Length (m)	4.732
Period (mm)	56
Number of periods	84.5
Minimum gap (mm)	20
Energy range (eV)	200–3000 (LH)
	440–3000 (LV)
	330–1500 (C)

**Table 4:** List of all gratings in the I21 PGM before the sample position Note that for VPG4, which has two coating stripes (Pt and Au), any parameters with multiple values represent the measurement performed with different coating stripes.

Beamline grating	VPG1	VPG2	VPG3	VPG4
Ruled area (mm)	160 × 30	160 × 30	160 × 30	160 × 30
Blaze angle (°)	0.62	–	–	0.80 (Pt)
				0.76 (Au)
Apex angle (°)	174.4	–	–	175.9 (Pt)
				176.1 (Au)
Groove width-to-period ratio	–	0.66	0.62	–
Groove depth (nm)	–	8.6	5.9	–
Coating thickness (nm)	33 (Au)	24 (Au)	25 (Au)	24 (Au)
				28 (Pt)
Roughness (Å, r.m.s.)	1.80	–	–	0.11 (Au)
				0.28 (Pt)
Tangential slope error (µrad, r.m.s.)	0.175	0.163	0.090	0.163 (Au)
				0.135 (Pt)
*a*_0_ (l/mm)	600	999.755	2000.134	1199.624
*a*_1_ (l/mm^2^)	5.532 × 10^−5^	4.303 × 10^−5^	3.815 × 10^−5^	3.82 × 10^−5^
*a*_2_ (l/mm^3^)	2.0 × 10^−9^	1.151 × 10^−9^	1.174 × 10^−9^	2.49 × 10^−9^
*a*_3_ (l/mm^4^)	1.1 × 10^−11^	1.836 × 10^−12^	3.674 × 10^−12^	2.17 × 10^−12^
Manufacturer	Zeiss and HZB	Zeiss and Shimadzu	Zeiss and Shimadzu	Zeiss and HZB

**Table 5:** List of all gratings in the I21 SGM after the sample position

Spectrometer grating	SVLS1	SVLS2	SVLS3
Ruled area (mm)	190 × 30	190 × 30	160 × 28.7
Blaze angle (°)	–	–	0.68
Apex angle (°)	–	–	176.6
Groove width-to-period ratio	0.68	0.60	–
Groove depth (nm)	7.8	5.24	–
Coating thickness (nm)	30 (Au)	30 (Pt)	30 (Pt)
Roughness (Å, r.m.s.)	1.80	0.25	0.167
Substrate material	Fused silica	Fused silica	Silicon
Tangential slope error (µrad, r.m.s.)	0.136	0.090	0.101
*a*_0_ (l/mm)	1500.286	2699.6	1500.017
*a*_1_ (l/mm^2^)	7.564 × 10^−2^	0.1584	−4.522 × 10^−2^
*a*_2_ (l/mm^3^)	−1.139 × 10^−4^	−1.881 × 10^−4^	−4.123 × 10^−4^
*a*_3_ (l/mm^4^)	5.528 × 10^−8^	1.14 × 10^−7^	3.4 × 10^−7^
Tangential radius (m)	94.51	94.36	115.8004
Manufacturer	JTEC and Shimadzu	JTEC and HORIBA	JTEC and HZB

**Table 6:** List of manipulator motion axes with corresponding motion specifications

Axis	Range	Minimum incremental step	Unidirectional reproducibility
*x*	±5 mm	<5 µm	0.5 µm
*y*	±5 mm	<5 µm	0.5 µm
*z*	±50 mm	<1 µm	0.5 µm
θ	>345°	<0.001°	<0.001°
χ	−30° → +45°	<0.05°	<0.05°
ϕ	±170°	<0.01°	<0.05°

## Appendix A VLS grating focusing

We show below the grating diffraction formula, the expressions of the VLS grating focal condition, the coma aberration and the spherical aberration (Strocov *et al.*, 2011 ▸; Chiuzbăian *et al.*, 2012 ▸):

In the above, α and β are X-rays incident and diffraction angles of grating optics, respectively. *R* is the radius of curvature of the gratings (∼∞ for the VPG at the beamline). λ is the wavelength. *r*_1_ is the distance from the photon source to the VPG grating, and *r*_2_ is distance from the VPG grating to the exit slit. *k* is the diffraction order. *a*_*i*_ are coefficients of a VLS grating with local groove density (Strocov *et al.*, 2011 ▸)

All the above equations are applicable to the spectrometer SVLS grating optics. In this case, *R* is the actual radius of curvature of the SVLS gratings. *r*_1_ is the distance from the sample vertical focal beam spot to the SVLS grating, and *r*_2_ is the distance from the SVLS grating to the CCD detector.

## Appendix B Energy resolution of the beamline

Below we show the individual contribution, namely, the photon source, the PGM optics and the exit slit, to the beamline energy resolution (Strocov *et al.*, 2011 ▸):

in which *E* is the X-ray energy, Σ_S_, δ_Gr_, δ_PM_ and Σ_Ex_ represent the full width at half-maximum (FWHM) of the vertical photon source size, the tangential slope errors of the VLS grating, the tangential slope errors of the M3 pre-mirror and the exit slit vertical opening, respectively. *r*_1_ and *r*_2_ stand, respectively, for the length of the entrance arm and the length of the exit arm with respect to the PGM grating optics. *a*_0_, *m* and λ are the constant line density of the VPG grating, the diffraction order (equal to 1) and the X-ray photon wavelength, respectively. α and β are the incident and diffraction angle (with respect to the normal) of the VPG grating.

The primary beamline energy resolution is a quadratic sum of the four contributions:
